# Pulmonary Edema and Stunned Myocardium in Subarachnoid Hemorrhage

**DOI:** 10.7759/cureus.7746

**Published:** 2020-04-20

**Authors:** Ryan Naum, Asia Filatov, Kettia Alusma-Hibbert, Patricio S Espinosa

**Affiliations:** 1 Osteopathy, West Virginia School of Osteopathic Medicine, Lewisburg, USA; 2 Neurology, Charles E. Schmidt College of Medicine, Florida Atlantic University, Boca Raton, USA; 3 Neurology, Marcus Neuroscience Institute, Boca Raton Regional Hospital, Boca Raton, USA

**Keywords:** subarachnoid, hemorrhage, brain bleed, pulmonary edema, cardiomyopathy, stress-related cardiomyopathy, intracranial hemorrhage, headache, complicated subarachnoid hemorrhage, takotsubo

## Abstract

Aneurysmal subarachnoid hemorrhage is a life-threatening event that can cause permanent disability. This life-threatening event can be further complicated by subsequent cardiac and pulmonary disability. The presence of a neurogenic cardiomyopathy and pulmonary edema increases the morbidity and mortality of patients who suffer from aneurysmal subarachnoid hemorrhage.

In this paper, we discuss a 39-year-old woman who presented to the emergency department (ED) with a chief complaint of a pounding headache with associated nausea and vomiting for the past three days. She had a past medical history significant only for migraines. During her stay in the ED, she began to exhibit signs of altered consciousness, hemoptysis, and respiratory compromise. Neuroimaging showed evidence of subarachnoid hemorrhage. The exact source of her subarachnoid hemorrhage could not be located with neuroimaging or angiography. Her clinical course was complicated by pulmonary edema and neurogenic stunned myocardium, and is still ongoing.

## Introduction

Aneurysmal subarachnoid hemorrhage can cause severe disability and is highly fatal. It can be further complicated by the presence of pulmonary edema and cardiac manifestations, both of which directly indicate the severity of the hemorrhage, as well as the overall prognosis [1]. Patients can develop Takotsubo-like cardiomyopathy and neurogenic pulmonary edema as a result of neurogenically induced overstimulation of the sympathetic nervous system via the brain-heart connection and may complicate poor grade aneurysmal subarachnoid hemorrhage [2].

## Case presentation

A 39-year-old woman presented to the emergency department (ED) with a chief complaint of a pounding headache with associated nausea and vomiting for the past three days. Her last known normal was four days prior to arrival. She stated that three days prior to arrival, she had a sudden onset of a pounding headache. She had never had a headache like this before. She stated that she took aspirin for the headache, which provided no relief. She denied any loss of consciousness, loss of sensation, loss of motor function, difficulty speaking, facial drooping, or loss/change in vision. She described her headache as constant and pounding. She also complained of associated nausea, and on the day of admission had been vomiting profusely. She was accompanied by her mother at bedside who provided a lot of the patient’s history. One day prior to her admission, she presented at another facility with the same complaints. No imaging was performed. She was given a butalbital compound for a presumed diagnosis of migraine, which offered minimal relief. 

Per her mother, she has a past medical history of migraines, heart palpitations, anxiety, and panic attacks. She takes no daily medications. She has a family history of a ruptured intracranial aneurysm in her father. Two months prior to presentation, she traveled to Colombia for vacation.

**Figure 1:**
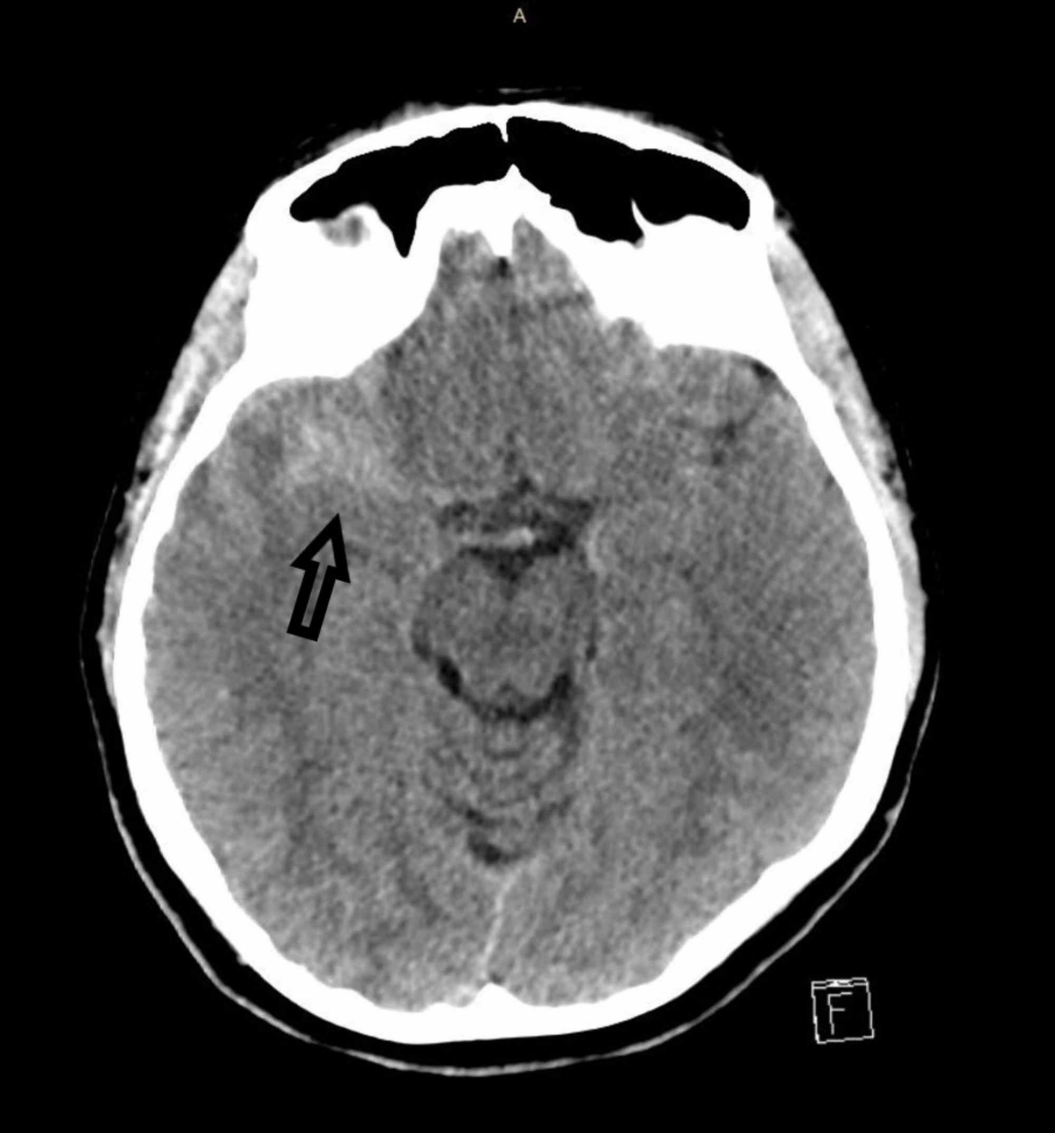
CT of the brain: axial view An axial view of a CT brain showing blood along the right Sylvian fissure.

**Figure 2:**
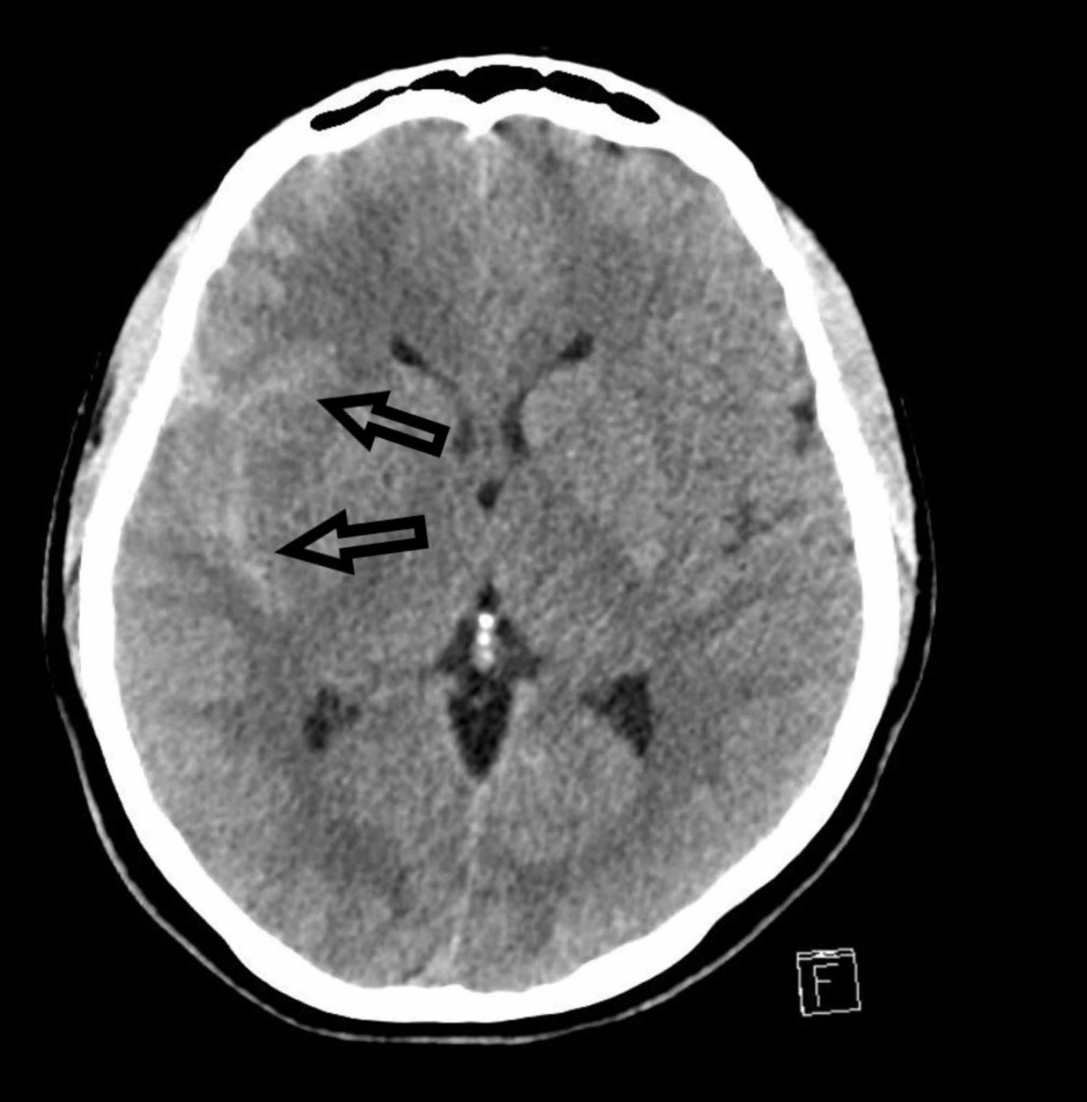
CT of the brain: axial view A CT of the brain showing blood along the Sylvian fissure. This image is more cephalad than the image shown in Figure 1.

At 3 am, on the morning of presentation to our facility, she called her mother, stating that the headache is back, and requested that she take her to the emergency room. While in the ED, she underwent a computed tomography (CT) scan without contrast of her brain. Imaging revealed subarachnoid blood along the right Sylvian fissure, as well as right frontal convexity, suggestive of a ruptured berry aneurysm (see Figures 1, 2). Based on imaging, she had a Hunt and Hess score of +4 and a Fisher score of 2. A stroke alert was subsequently called. Her last known normal was four days prior. She had an initial National Institute of Health (NIH) stroke score of 0 upon presentation to the ED. In the ED, the patient was hypertensive, with a systolic blood pressure of 190 mmHg, tachycardic with a heart rate ranging from 120 to 160 beats per minute, and tachypneic with a respiratory rate of 16-40 breaths per minute. A CT angiogram was performed, but showed no acute abnormalities. Initial labs indicated leukocytosis and lactic acidosis, meeting the systemic inflammatory response syndrome (SIRS) criteria. She was started on a Cardene drip for blood pressure management, labetalol for fine tuning of blood pressure, levetiracetam for seizure prophylaxis, Crestor for neuroprotection, and pantoprazole for nausea. 

On physical exam, the patient appeared to be in acute distress. She was diaphoretic with agonal breathing. She appeared to be lethargic, but was able to answer questioning. She was oriented to person, place, and time, and able to provide an accurate history. There were no signs of dysarthria, and she had normal comprehension and fluency. Her pupils were equal, round, and reactive to light and accommodation. She was able to smoothly track objects in all directions with her eyes with no signs of nystagmus. Her face was symmetric with equal and symmetric eyebrow raise and smile bilaterally. Sternocleidomastoid muscle and trapezius muscle functions were intact bilaterally. She had normal muscle bulk and tone in both the upper and lower extremities bilaterally. Deltoid, grip, biceps, and triceps muscle strength was 3/5 bilaterally. Hip flexor and extensor muscle strength was 3/5 bilaterally. Ankle dorsiflexor and plantarflexor muscle strength was 3/5 bilaterally. Biceps, triceps, and brachioradialis deep tendon reflexes (DTRs) were all 2+ bilaterally. Patellar and Achilles DTRs were also 2+ bilaterally. Sensory examination, coordination, and gait were unable to be evaluated due to the patient’s rapidly declining state while in the ED.

**Figure 3:**
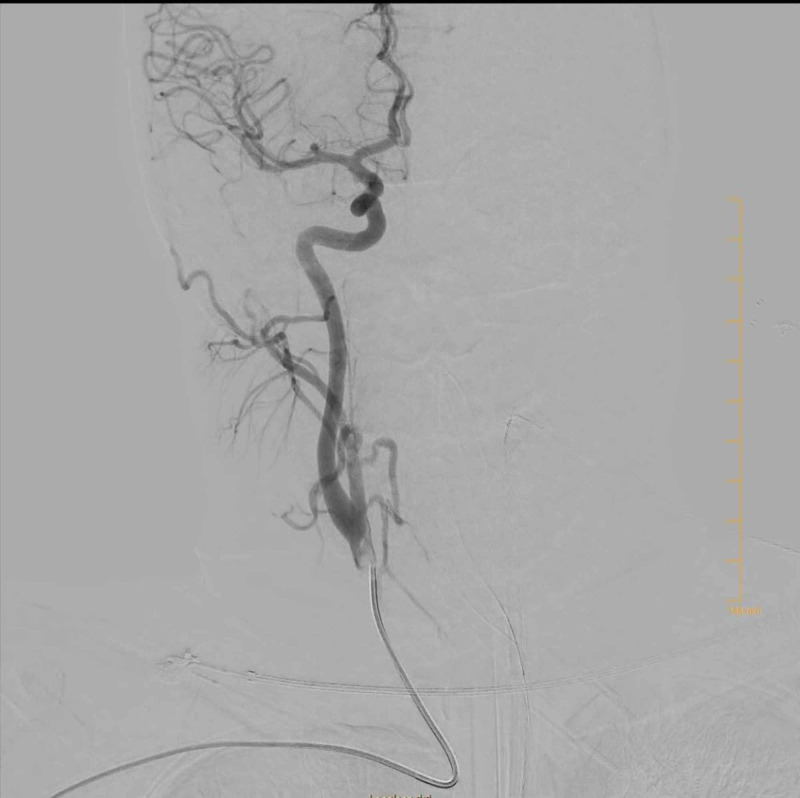
Angiogram of right carotid artery Angiogram of the right carotid artery showing no vascular abnormalities of berry aneurysm.

While in the ED, the patient began to decompensate, and was unable to protect her airways. She was promptly intubated. Neurosurgery was consulted and a right ventricular shunt was placed. The patient then underwent a cerebral angiogram. Cerebral angiogram showed no vascular abnormalities that could have caused the patient’s subarachnoid hemorrhage localized within the horizontal and vertical portions of the right Sylvian fissure (seen in Figure 3). Cerebral angiography was completed three additional times. All results were negative. No source of bleeding could be determined and no aneurysm could be identified. No intervention was performed. A transcranial Doppler showed no evidence of spasm or occlusion. 

**Figure 4:**
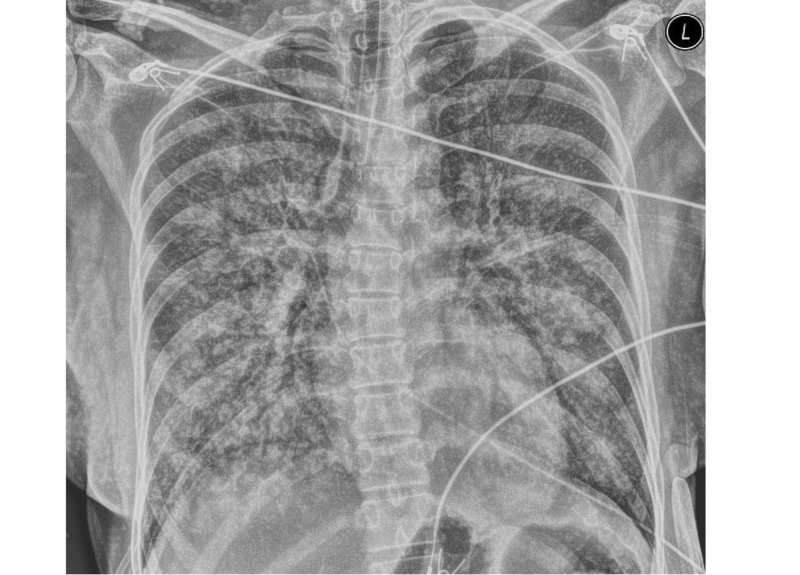
Chest X-ray A chest X-ray showing diffuse infiltrates consistent with cardiopulmonary edema or acute respiratory distress syndrome.

The patient was transferred to the neurointensive care unit (neuro ICU) following the cerebral angiogram. She was noted to have progressive decline in her respiratory status on the ventilator. Refractory hypoxemia developed. Chest x-ray was obtained (Figure 4), showing diffuse infiltrates consistent with cardiopulmonary edema or acute respiratory distress syndrome.

**Figure 5:**
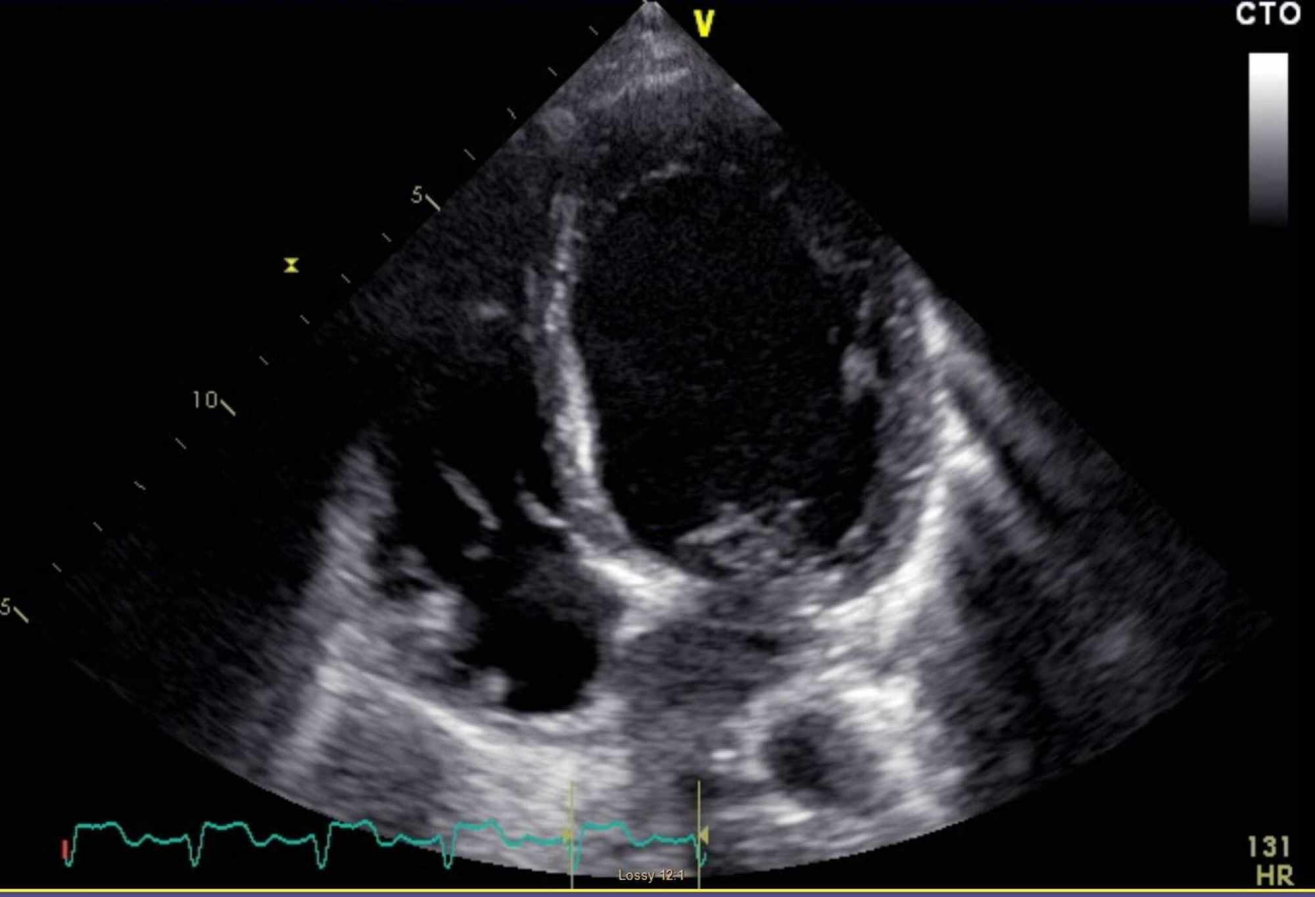
Transthoracic echocardiogram There is evidence of a severely dilated left ventricle with a measured ejection fraction of 15%.

A bedside transthoracic echocardiogram was performed, revealing a severely dilated left ventricle with an ejection fraction of 15% (Figure 5). Her clinical course is still ongoing. 

## Discussion

Subarachnoid hemorrhage refers to bleeding in the subarachnoid space, which lies between the arachnoid and pia mater. This space is typically filled with cerebrospinal fluid. Most cases of subarachnoid hemorrhage are caused by rupture of an intracranial aneurysm. Approximately 15% to 20% of patients presenting with subarachnoid hemorrhage do not have vascular lesion on initial four-vessel cerebral angiography. Patients with subarachnoid hemorrhage will typically present with a sudden and severe onset of headache, typically described as a "thunderclap headache". Patients may describe the headache as "the worst headache of my life". Patients may also present with a brief loss of consciousness, vomiting, and neck pain or stiffness [3]. Physical exam often shows hypertension and may show meningismus. Terson syndrome (preretinal hemorrhages) may be seen and implies a poorer prognosis [4].

A detailed physical and neurological exam is necessary for the evaluation of subarachnoid hemorrhage. Physicians should have a high clinical suspicion in patients who present with a sudden or rapid onset of severe headache. A non-contrast head CT should be obtained, followed by a lumbar puncture if the head CT is negative. Blood is generally found in the basal cisterns on head CT, but can also be seen in the area of the Sylvian fissures, interhemispheric fissure, interpeduncular fossa, and suprasellar, ambient, and quadrigeminal cisterns. If head CT is negative, lumbar puncture should be performed, and is likely to show an elevated opening pressure, elevated red blood cell count that does not diminish from cerebrospinal fluid tube 1 to tube 4, and xanthochromia [5]. One of the most widely used grading systems for subarachnoid hemorrhage severity is the Hunt and Hess grading system. Table 1 outlines the Hunt and Hess grading system [6].

**Table 1:** The Hunt and Hess grading system

Grade	Symptoms
1	Asymptomatic or mild headache and slight nuchal rigidity.
2	Moderate to severe headache, stiff neck, no neurological deficit except cranial nerve palsy.
3	Drowsy or confused, mild focal neurological deficit.
4	Stupor, moderate, or severe hemiparesis.
5	Deep coma, decerebrate posturing.

The Fisher scale is another grading system used to establish the risk of vasospasm based upon hemorrhage pattern seen on initial head CT scan. Table 2 outlines the Fisher scale [7]. Patients diagnosed with subarachnoid hemorrhage should be transferred to the neuro ICU for blood pressure control, maintenance of euvolemia, treatment with nimodipine, and continuous hemodynamic and neurological monitoring [8]. 

**Table 2:** The Fisher Scale

Group	Head CT Findings
1	No blood detected.
2	Diffuse deposition or thin layer with all vertical layers of blood (in interhemispheric fissure, insular cistern, or ambient cistern) less than 1 mm thick.
3	Localized clots and/or vertical layers of blood 1 mm or more in thickness.
4	Intracerebral or intraventricular clots with diffuse or no subarachnoid blood.

Aneurysmal subarachnoid hemorrhage causes permanent disability and is highly fatal. Pulmonary edema and cardiac manifestations indicate the severity of subarachnoid hemorrhage. The overall prognosis depends on the degree of delayed cerebral ischemia, volume of the initial bleed, and re-bleeding [1]. Aneurysmal subarachnoid hemorrhage may be accompanied by acute cardiopulmonary complications, Takotsubo-like cardiomyopathy, and neurogenic pulmonary edema. The above dysfunctions appear to result from a neurogenically induced overstimulation of the sympathetic nervous system via the brain-heart connection and may complicate poor-grade aneurysmal subarachnoid hemorrhage [2]. Takotsubo-like cardiomyopathy increases the mortality rate of subarachnoid hemorrhage [9]. Similarly, Garg and Bar state that various systemic complications occur as a result of aneurysmal subarachnoid hemorrhage. Complications of aneurysmal subarachnoid hemorrhage include electrocardiographic changes, troponin elevation, neurogenic pulmonary edema, anemia, hyponatremia, and neurogenic stunned myocardium [10]. 

A stunned myocardium is defined as a state in which a given section of the myocardium manifests with a certain form of contractile abnormality. Similarly, a neurogenic stunned myocardium refers to cardiac abnormalities that occur as a result of neurological disorders, such as seizures, stroke, etc [11]. A neurogenic stunned myocardium occurs secondary to reversible posterior leukoencephalopathy, seizures, subarachnoid bleed, Guillain-Barre syndrome, stroke, and/or brain tumor [12]. Neurological events such as seizures and stroke in neurogenic stunned myocardium may lead to myocardial injury [13]. These events result in autonomic nervous system dysfunction and cause myocardial injury. Clinical features associated with neurogenic stunned myocardium include left ventricular dysfunction, elevated troponin level, and changes on the electrocardiogram [11]. Stunned myocardium is characterized by reversible left ventricular dysfunction and may be caused by a subarachnoid hemorrhage. Neurogenic pathologies may lead to complications such as prolonged intubation, arrhythmias, and pulmonary edema, which may negatively impact long-term recovery from subarachnoid hemorrhage and increase mortality and morbidity [14]. 

Pulmonary edema may complicate subarachnoid hemorrhage. Patients who manifest with poor-grade subarachnoid hemorrhage are almost always complicated with cardiopulmonary dysfunctions, especially Takosubo-like cardiomyopathy and neurogenic pulmonary edema. Subarachnoid hemorrhage involves massive catecholamine release into circulation, following aneurysm rupture, and is responsible for increased vascular permeability, endothelial damage, and vasoconstriction [15]. Neurogenic pulmonary edema is characterized by acute respiratory-distress elicited by acute/severe compromise of the central nervous system. Neurogenic pulmonary edema is diagnosed by the presence of pink-frothy sputum, PaO_2_:PiO_2_ <200 mmHg, pulmonary edema, bilateral opacities on X-ray, the absence of alternative causes of respiratory illness, and rapid resolution within 48 to 72 hours. The most common causes of neurogenic pulmonary edema include subarachnoid bleeding, enterovirus-71-associated brain-stem encephalitis, traumatic brain injury, epilepsy, intracranial injury, multiple sclerosis, subarachnoid bleeding, electroconvulsive therapy, intracranial/spinal surgery, intoxication, and hypoxia [16]. 

## Conclusions

Aneurysmal subarachnoid hemorrhage is usually caused by the rupture of an intracerebral aneurysm, but in approximately 15% to 20% of cases the cause is not known. This life-threatening event can be further complicated by subsequent cardiac and pulmonary disability. The cause of this disability is likely from an overstimulation of sympathetic pathways, leading to a stunned myocardium and neurogenic pulmonary edema. The goal of care should focus on maintaining euvolemic status, treatment with nimodipine, and continuous hemodynamic and neurological monitoring.
